# Longitudinal study on the mental health trajectory of new graduate nurses during role transition: identifying critical windows and core influencing factors

**DOI:** 10.3389/fpubh.2026.1795005

**Published:** 2026-04-09

**Authors:** Qian Su, Rui Wang, Xiaolu Wei, Yanfei Hu, Lin Han

**Affiliations:** 1 School of Nursing, Lanzhou University, Lanzhou, Gansu, China; 2 Nursing Department, Gansu Provincial Hospital, Lanzhou, Gansu, China

**Keywords:** critical windows, longitudinal study, mental health, new graduate nurses, trajectory

## Abstract

**Objective:**

This study aimed to establish a dynamic follow-up cohort of new graduate nurses to systematically explore the dynamic changes and developmental trajectory of their mental health status within 3 years after employment. It further analyzed differences in mental health at different time points and factors associated with these trajectories, with the goal of providing empirical evidence for early identification of psychological risks and implementing targeted psychological interventions. This would assist new graduate nurses in successfully navigating the role transition period.

**Methods:**

A prospective longitudinal study using a dynamic cohort design was conducted from 2019 to 2024. A total of 130 new graduate nurses were recruited across three cohorts; of these, 118 completed the baseline assessment. Follow-up assessments were completed by 115 (97.5% of baseline) at year one, 115 (97.5%) at year two, and 102 (86.4%) at year three. Overall, 88 participants (74.6% of those with baseline data) completed all four assessments. A self-designed general information questionnaire and the Symptom Checklist-90 (SCL-90) were used to assess mental health status across 10 dimensions. A linear mixed-effects model was employed to examine longitudinal trends and factors associated with mental health trajectories. Time was modeled as a continuous variable (coded as 0, 1, 2, 3) to estimate longitudinal associations.

**Results:**

Over the 3-year period after employment, the mental health status of new graduate nurses showed a gradual decline as their working time increased. Among the symptoms, obsessive-compulsive states were the most prominent. The linear mixed-effects model revealed statistically significant differences (*P* < 0.05) in scores across seven dimensions: somatization, depression, anxiety, fear, hostility, psychoticism, and eating/sleep problems. Further analysis indicated that new graduate nurses who fell asleep quickly with better sleep quality, worked no night shifts, and held the title of “nurse” had significantly better mental health than those who experienced difficulty falling asleep with poor sleep quality, worked night shifts, and held the title of “senior nurse” (β = 11.62; β = −7.72; β = 7.23; β = 12.12).

**Conclusion:**

This longitudinal study finds that while new graduate nurses' overall mental health remains acceptable within 3 years, it declines significantly with experience. The trajectory evolves in stages: a “high-pressure role adaptation phase,” a “plateau phase of adaptation decline,” and a “career development confusion phase,” with issues especially in somatization, anxiety, and depression. Key associated factors include sleep quality, time to fall asleep, night-shift frequency, and professional title. Nursing managers should monitor these dynamic changes and implement early interventions—such as better sleep management, optimized scheduling, and phased psychological support—to facilitate successful role adaptation.

## 1 Introduction

With the continuous improvement of China's healthcare service system, the nursing workforce is increasingly trending toward younger demographics. Approximately 300,000 new graduate nurses join the clinical workforce annually (1). As fresh additions and crucial future reserves for hospital nursing teams, the professional development and psychological adaptation of new graduate nurses directly impact the quality of healthcare services and the sustainable development of medical institutions (2). According to China's *Guidelines for New Nurse Orientation Training (Trial)*, new graduate nurses—defined as registered nurses in their first year of practice with no prior work experience—must undergo at least 2 years of standardized training after employment to complete the transition from student to professional nurse, a phase defined as the role transition period [(3) p. 25]. In this study, new graduate nurses specifically refer to nurses who: (a) graduated within 12 months prior to employment, (b) hold a Registered Nurse (RN) license (the sole nursing registration level in China, equivalent to RN in North America), and (c) have no prior paid nursing work experience. This distinguishes them from “new hires with experience” or Licensed Practical Nurses (LPNs), a role that does not exist in China's nursing system. However, due to multiple stressors such as adapting to a new environment, lack of experience, and the gap between theory and practice, new graduate nurses are prone to experience **Transition Shock** (4) during this process, leading to a series of psychological issues including anxiety and occupational stress (4, 5). If not effectively addressed over the long term, this can ultimately result in severe consequences such as turnover intention (6–8).

The role transition period is a dynamic, critical, and indispensable stage in a nurse's career development. According to Meleis's Transition Theory, role transition is not a singular event but a process involving phases of entry, passage, and exit, during which individuals experience varying degrees of stress and adaptation (9). Similarly, Selye's General Adaptation Syndrome posits that prolonged exposure to stressors leads to distinct stages of response: alarm, resistance, and exhaustion (10). Assessing their mental health status during this phase should not rely on a static, “all-or-nothing” perspective but should instead focus on the trajectory of their psychological changes over time. Existing cross-sectional studies, while indicating a high prevalence of psychological distress among new graduate nurses (11, 12), struggle to reveal the dynamic evolution of their mental state or distinguish between short-term adaptive stress and persistent psychological problems. This is particularly relevant for new graduate nurses as an early-career group, whose mental health may exhibit unique developmental patterns that align with theoretical models of stress adaptation (13). Drawing on these theoretical frameworks, new graduate nurses' mental health may follow distinct patterns during their role transition. However, most existing studies have used cross-sectional designs, which cannot capture such dynamic changes. This study therefore uses an exploratory longitudinal design to examine how new graduate nurses' mental health actually changes over time and whether identifiable phases emerge during the first 3 years of employment. If such phases exist, they may represent critical windows for intervention—periods when nurses are particularly vulnerable or especially receptive to support. Failure to accurately understand the trajectory of their mental health and identify key turning points during the initial career stage will hinder nursing managers from implementing precise and effective psychological support measures during optimal intervention windows.

Therefore, by establishing a dynamic follow-up cohort of new graduate nurses, this study aims to systematically explore the dynamic changes and developmental trajectory of their mental health status within 3 years after employment. It further seeks to analyze differences in mental health at different time points and factors associated with these trajectories. The goal is to provide empirical evidence for the early identification of psychological risks and the implementation of staged, targeted psychological interventions, thereby supporting new graduate nurses in successfully navigating the role transition period.

## 2 Methods

### 2.1 Design

This study employed a prospective longitudinal research design. Starting in 2019, a prospective dynamic cohort of new graduate nurses was established at a comprehensive tertiary hospital in Lanzhou City, Gansu Province. New graduate nurses meeting the inclusion criteria were followed for a period of 3 years.

### 2.2 Participants

Using cluster sampling, all new graduate nurses meeting the inclusion criteria were enrolled annually. Recruitment Process: Upon commencing employment, eligible new graduate nurses were invited to participate during their orientation week. Investigators explained the study purpose, procedures, and confidentiality measures. Written informed consent was obtained from all participants. Participation was entirely voluntary, with no incentives provided, and participants could withdraw at any time without penalty. Inclusion Criteria: (a) Possession of a valid nursing license; (b) Recent nursing graduates with no prior work experience (excluding clinical internships with no prior work experience (excluding clinical internships); (c) Informed consent and voluntary participation in this study. Exclusion Criteria: (a) History of depression, anxiety, or other psychiatric disorders; (b) Poor compliance. Withdrawal Criteria: (a) Subjects who voluntarily withdraw and no longer wish to participate in the study; (b) Subjects who, during the study period, are unable to complete the survey or data collection due to various reasons (e.g., force majeure).

### 2.3 Instruments

#### 2.3.1 Demographic questionnaire

Designed by the research team, the study collected demographic, lifestyle, and work-related characteristics of the research subjects: sex, educational background, marital status, sleeping patterns, sleep quality, physical activity, professional title, number of night shifts per month, rotating departments. Sex was recorded as male or female based on self-report; we acknowledge that a binary classification does not capture the full spectrum of sex or gender diversity.

#### 2.3.2 Symptom checklist-90 (SCL-90)

One of the most renowned mental health assessment tools, the Symptom Checklist-90 (SCL-90), was developed by Derogatis and colleagues (14). It encompasses a broad spectrum of psychopathological symptoms and accurately reflects subjective symptom characteristics across diverse populations. This scale has found extensive application in clinical psychology, mental health services, and psychological counseling. It comprises 10 dimensions (somatization, obsessive-compulsive, interpersonal sensitivity, depression, anxiety, hostility, phobic anxiety, paranoid ideation, psychoticism, and others) with 90 items. Using a 5-point Likert scale (1 = no symptoms, 5 = severe symptoms), the total score ranges from 0 to 450. Higher scores indicate poorer mental health among new graduate nurses. The Cronbach's α coefficients for the scale and its dimensions range from 0.77 to 0.90, demonstrating good reliability and validity (14).

### 2.4 Data collection

**Figure 1:**
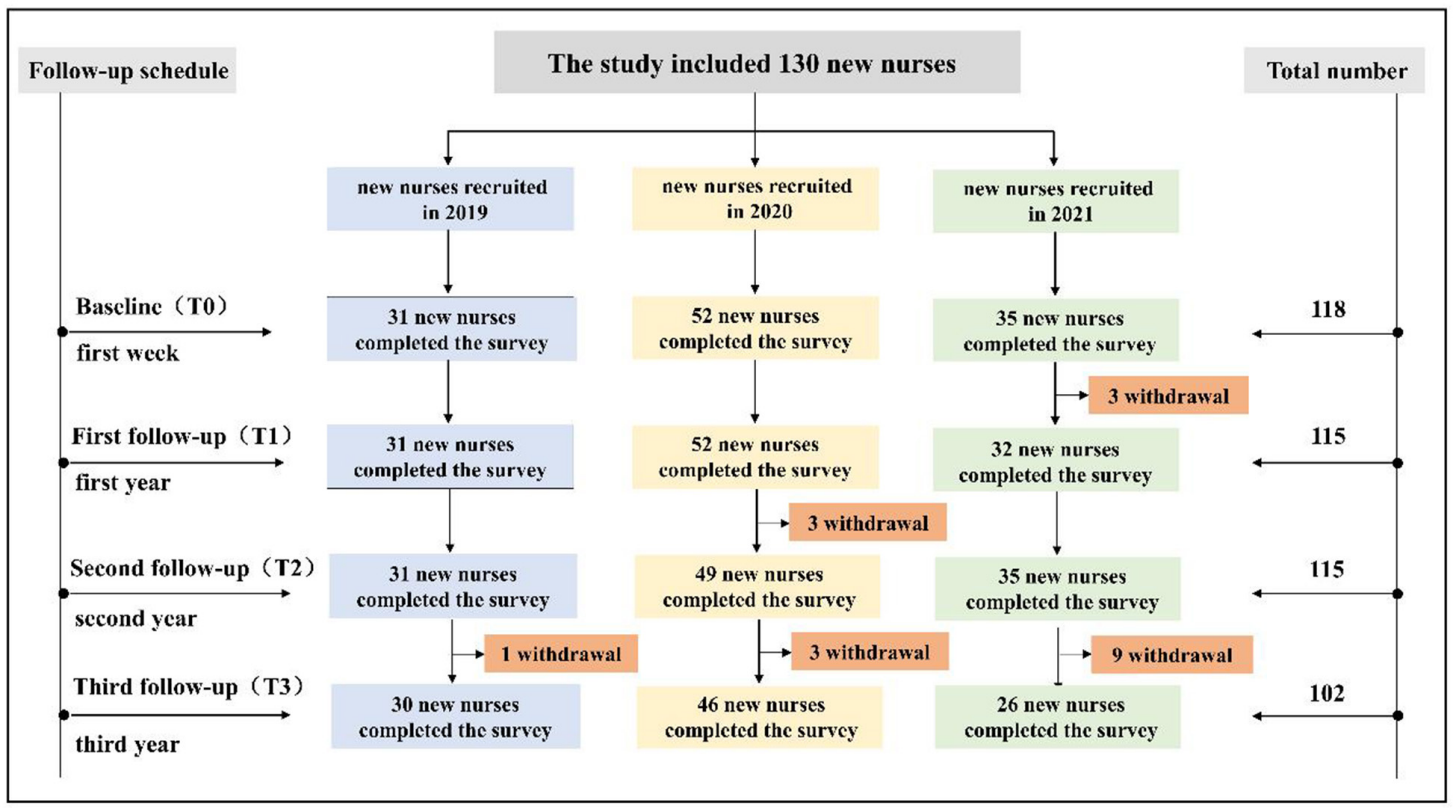
Flowchart of participant enrollment.

Data were collected using a questionnaire-based survey. Considering the substantial number of items across the scales used, paper-based questionnaires were administered to ensure data accuracy and overall response quality. This approach is supported by evidence that paper-based methods can produce higher overall mean scores, greater variability in responses, and higher positive affect for responding compared to web-based surveys, particularly for lengthy assessments (15). New graduate nurses were surveyed at four time points: the first week (baseline), first year, second year, and third year after employment. Investigators conducted the surveys in a one-on-one manner. Attrition during follow-up was primarily due to resignation or leave of absence (Figure 1).

### 2.5 Participant retention and missing data

Given the longitudinal nature of this study, participant attrition was anticipated. To minimize dropout, we maintained regular contact with participants between follow-ups and used consistent data collection methods at each time point. A total of 130 new graduate nurses were recruited across three cohorts. At the baseline (T0) assessment, 118 nurses participated; the remaining 12 nurses were hired later and had their first assessment at T1, T2, or T3. Among the 118 nurses with T0 data, 88 completed all four assessments, while 30 missed at least one follow-up (16 did not attend T3, and 14 attended T3 but missed an intermediate wave). Reasons for missing assessments included resignation, leave of absence, and pregnancy. Missing data were assumed to be missing at random (MAR), as these reasons appeared to be unrelated to participants' mental health scores at the time of dropout. The linear mixed-effects model used in the analysis is appropriate for handling missing data under the MAR assumption and includes all available observations without listwise deletion.

### 2.6 Data analysis

Statistical analyses were performed using SPSS 27.0 and R Studio. Descriptive statistics summarized participant characteristics and SCL-90 scores. Repeated-measures ANOVA assessed differences in scores across time points for the total SCL-90 score and, on an exploratory basis, for each subscale; no adjustment for multiple comparisons was applied. To identify factors associated with mental health changes, a linear mixed-effects model (LMM) was utilized, with the total SCL-90 score as the dependent variable. Time-varying covariates—including night-shift frequency, professional title, and sleep characteristics (sleeping patterns and sleep quality)—were entered into the model using the values measured at each corresponding time point. All covariates were modeled using observed values at each wave; baseline values were not carried forward. Statistical significance was set at *P* < 0.05. The linear mixed-effects model handles missing data under the missing at random (MAR) assumption, using all available observations without excluding participants with incomplete follow-up data.

## 3 Results

### 3.1 Characteristics of the participants

Of the 130 recruited nurses, 118 provided baseline (T0) data. Among these, 88 participants completed all four assessments, and 30 missed at least one follow-up. An additional 12 nurses, who were hired later, had their first assessment at T1, T2, or T3 and were included in the longitudinal analyses. Table 1 presents the demographic and occupational characteristics of participants at each time point.

**Table 1:** Characteristics of the participants.

Variable	T0 (*n* = 118)		T1 (*n* = 115)		T2 (*n* = 115)		T3 (*n* =102)	
	*n*	%	*n*	%	*n*	%	*n*	%
Sex	
Male	11	9.32	11	9.57	10	8.70	10	9.80
Female	107	90.68	104	90.43	105	91.30	92	90.20
Educational background	
Bachelor's degree	115	97.46	111	96.52	110	95.65	97	95.10
Master's degree	3	2.54	4	3.48	5	4.35	5	4.90
Only child	
No	104	88.14	101	87.83	100	86.96	87	85.29
Yes	14	11.86	14	12.17	15	13.04	15	14.71
Marital status	
Unmarried	118	100.00	114	99.13	112	97.39	80	78.43
Married	0	0.00	1	0.87	3	2.61	22	21.57
Professional title	
Registered Nurse	112	94.92	110	95.65	66	57.39	26	25.49
Senior Nurse	6	5.08	5	4.35	46	40.00	72	70.59
Senior Charge Nurse	0	0	0	0	3	2.61	4	3.92
Number of night shifts per month	
0 days	118	100.00	52	45.22	21	18.26	11	10.78
1–5 days	0	0.00	45	39.13	59	51.30	57	55.88
6–10 days	0	0.00	17	14.78	34	29.56	33	32.35
>10 days	0	0.00	1	0.87	1	0.87	1	0.98
Sleeping patterns	
Fall asleep quickly	94	79.66	85	73.91	76	66.09	61	59.80
Difficulty falling asleep	18	15.25	24	20.87	28	24.35	30	29.41
Toss and turn	6	5.08	6	5.22	11	9.57	11	10.78
Sleep quality	
Difficult to wake up	34	28.81	33	28.70	38	33.04	38	37.25
Frequent nightmares	11	9.32	15	13.04	12	10.43	17	16.67
Easily awakened	73	61.86	67	58.26	65	56.52	47	46.08
Physical activity	
Almost never participate	25	21.19	19	16.52	37	32.17	47	46.08
Less than 4 times per week	69	58.47	85	73.91	65	56.52	40	39.22
4 or more Physical activity times per week	23	19.49	11	9.57	13	11.30	15	14.71
Rotating departments	
Internal medicine	21	17.80	33	28.70	26	22.61	30	29.41
Surgical department	32	27.12	33	28.70	40	34.78	31	30.39
Emergency room	9	7.63	6	5.22	7	6.09	2	1.96
ICU	11	9.32	9	7.83	5	4.35	9	8.82
Obstetrics and gynecology	9	7.63	5	4.35	8	6.96	4	3.92
Operating room	19	16.10	15	13.04	20	17.39	18	17.65
Others	17	14.41	14	12.17	9	7.83	8	7.84

Note: The professional titles examined in this study refer to China's Nursing Professional Title System. This system comprises five levels: Nurse (entry-level), Junior Nurse Practitioner (junior), Senior Nurse Practitioner (intermediate), Associate Chief Nurse Practitioner (associate senior), and Chief Nurse Practitioner (senior). Advancement requires meeting specific practice duration thresholds—varying by educational attainment—followed by passing a national unified examination. For bachelor's degree holders, who constituted 97.5% of this sample, the standard progression is: promotion to Junior Nurse Practitioner after 1 year of practice and successful examination; eligibility for Senior Nurse Practitioner examination after 4 years as Junior Nurse Practitioner. For the minority with master's degrees (2.5%), the pathway is accelerated: 1 year to Junior Nurse Practitioner, and only two additional years to Senior Nurse Practitioner eligibility. This tenure-based examination system differs fundamentally from Western Nurse Practitioner roles, which require graduate-level education rather than standardized tenure-based progression.

### 3.2 Mental health and its dimensional trajectories in new graduate nurses

As shown in Table 2, the total mental health scores and the scores of each dimension among new graduate nurses over their first 3 years of employment indicated that mental health status was poorest at the T3 time point. Among the symptoms, obsessive-compulsive state was the most prominent, with its highest score observed at T1.

**Table 2:** SCL-90 scores of new graduate nurses across multiple dimensions [Mean (SD)].

Item	T0	T1	T2	T3
Total score	122.03 (35.65)	131.00 (37.57)	129.57 (40.68)	138.70 (47.31)
Average score	1.36 (0.40)	1.46 (0.42)	1.44 (0.45)	1.54 (0.53)
Obsessive-compulsive	1.68 (0.53)	1.75 (0.54)	1.67 (0.61)	1.68 (0.65)
Interpersonal sensitivity	1.43 (0.52)	1.52 (0.49)	1.42 (0.51)	1.54 (0.59)
Paranoid ideation	1.40 (0.50)	1.46 (0.50)	1.45 (0.55)	1.49 (0.51)
Depression	1.35 (0.47)	1.45 (0.48)	1.47 (0.51)	1.55 (0.58)
Sleep/Eating disorders	1.34 (0.43)	1.51 (0.51)	1.49 (0.50)	1.47 (0.56)
Anxiety	1.31 (0.42)	1.38 (0.42)	1.41 (0.48)	1.53 (0.53)
Somatization	1.28 (0.41)	1.44 (0.46)	1.44 (0.47)	1.55 (0.54)
Psychoticism	1.28 (0.40)	1.35 (0.41)	1.33 (0.43)	1.43 (0.51)
Hostility	1.26 (0.37)	1.34 (0.40)	1.37 (0.48)	1.54 (0.65)
Phobic anxiety	1.15 (0.36)	1.27 (0.44)	1.28 (0.46)	1.36 (0.51)

**Figure 2:**
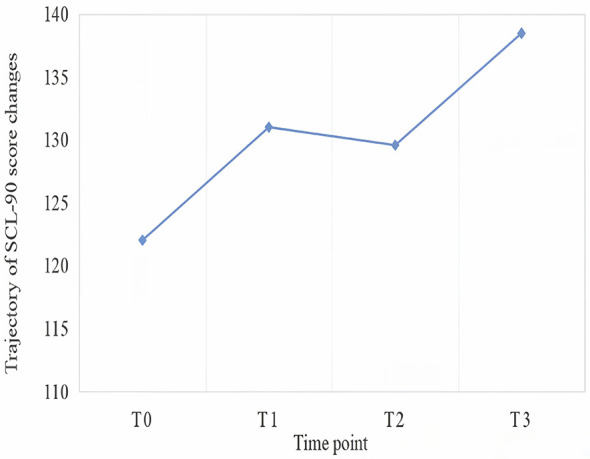
Trajectory of SCL-90 scores.

Figure 2 illustrates that the overall mental health status showed a deteriorating trend (i.e., higher scores indicating worse mental health) over the course of employment. A slight improvement (decrease in score) was noted at the T2 time point. Pairwise comparison among complete responders (*n* = 88) showed that the decrease from T1 to T2 was not statistically significant [mean difference = 0.01, 95% CI (−6.19, 6.22), *P* = 0.997; Supplementary Table 7].

**Figure 3:**
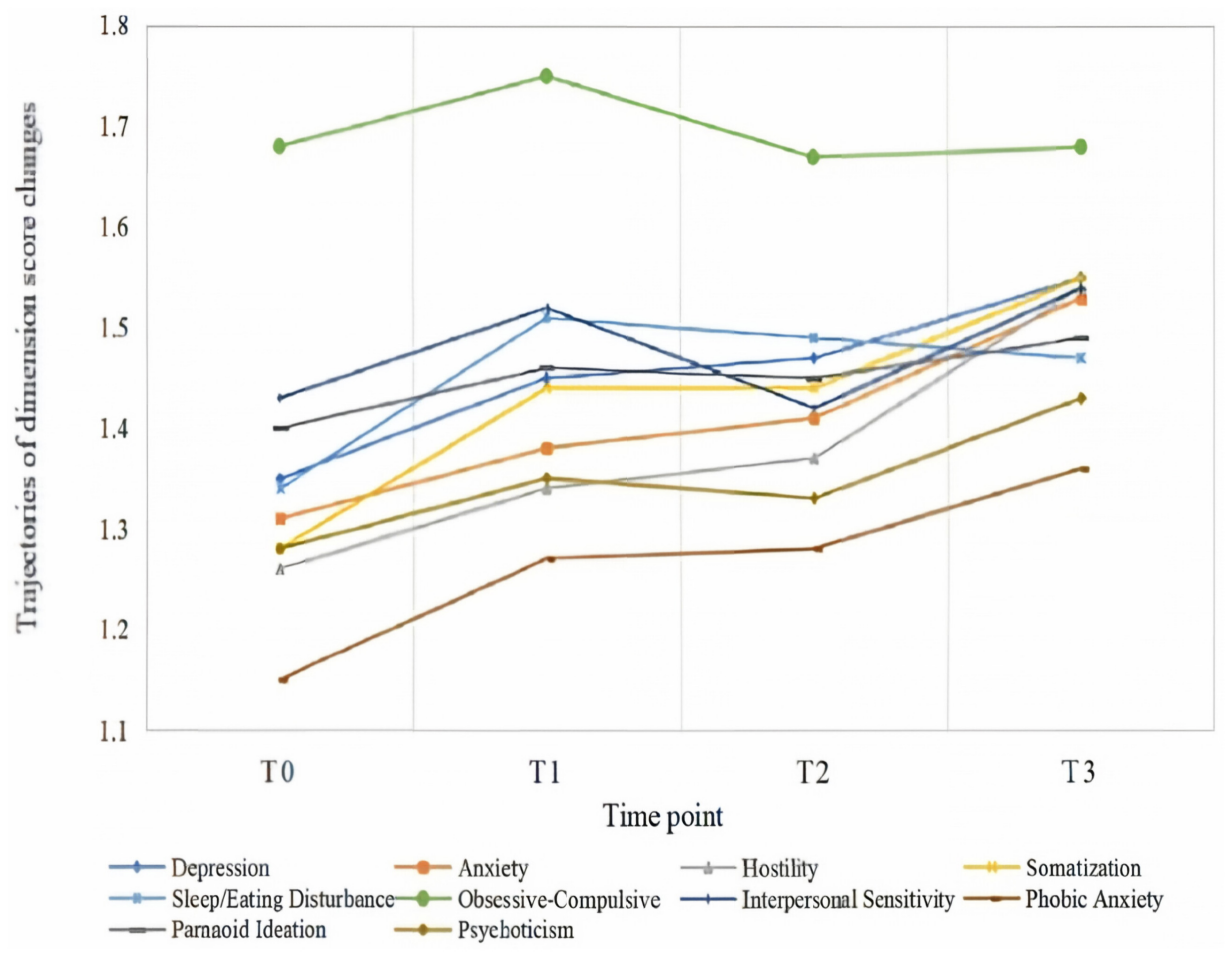
Trajectories of dimension scores changes.

Figure 3 presents the trends for each dimension. The scores for four dimensions—phobic anxiety, anxiety, depression, and hostility—demonstrated a steady upward trend. In contrast, the scores for the other five dimensions—interpersonal sensitivity, somatization, paranoid ideation, psychoticism, and issues related to diet/sleep—exhibited a fluctuating pattern of increase.

### 3.3 Linear mixed-effects analysis of mental health status in new graduate nurses

**Figure 4:**
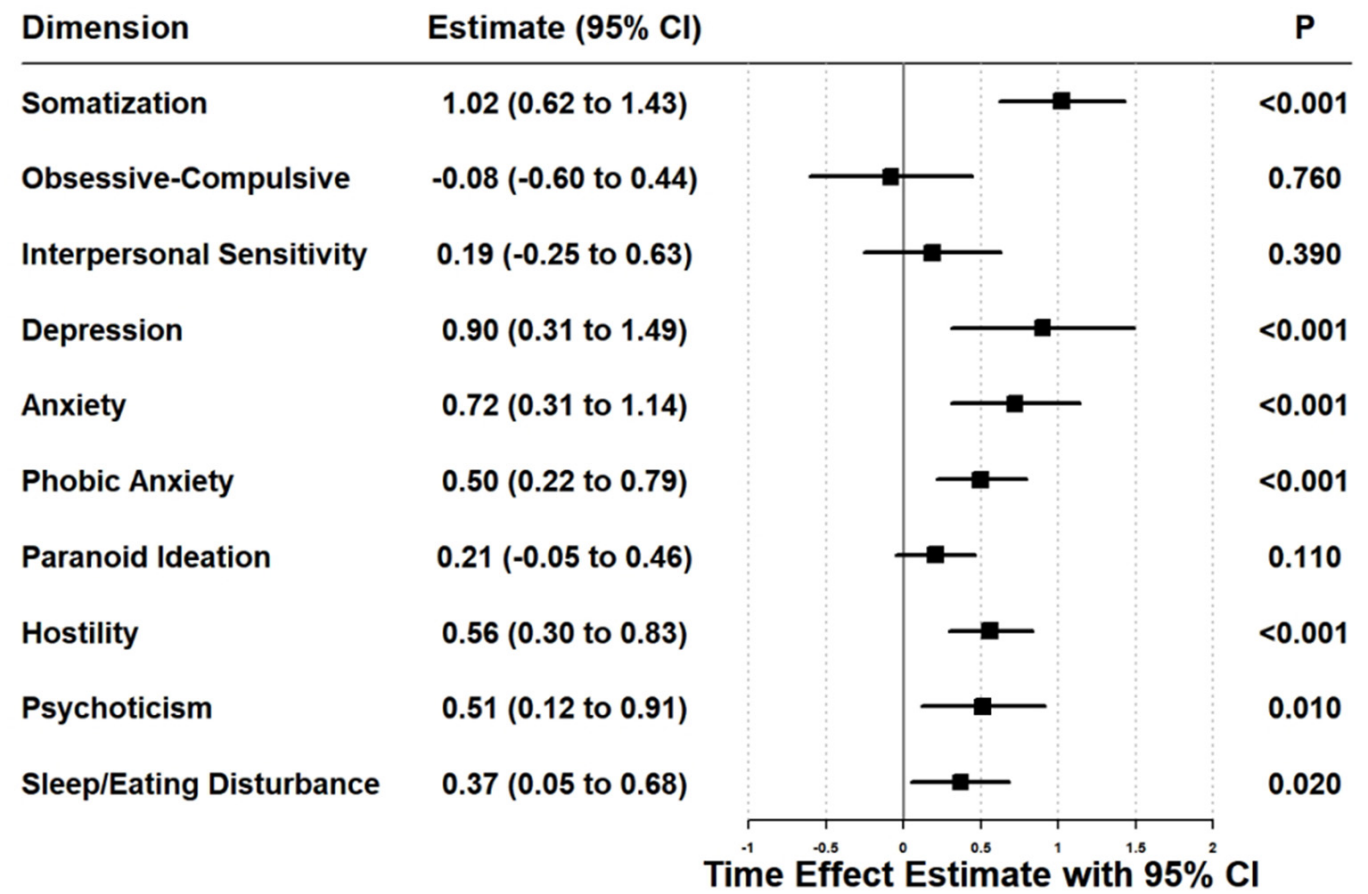
Linear mixed-effects model for mental health.

The results of the linear mixed-effects model are presented in Figure 4. A statistically significant increase in mental health scores (indicating worsening symptoms) was observed from T0 to T3 (Estimate = 5.35, *P* < 0.05). Furthermore, significant worsening trends were identified in seven specific dimensions: somatization, depression, anxiety, phobic anxiety, hostility, psychoticism, and sleep/appetite disorders (Figure 4).

### 3.4 Influencing factors of new graduate nurses' mental health

A linear mixed-effects model was used, with sex, only child, marital status, professional title, educational background, physical activity, rotating department, sleeping patterns, sleep quality, and year of employment as fixed effects, and individual nurse as random effects.

**Figure 5:**
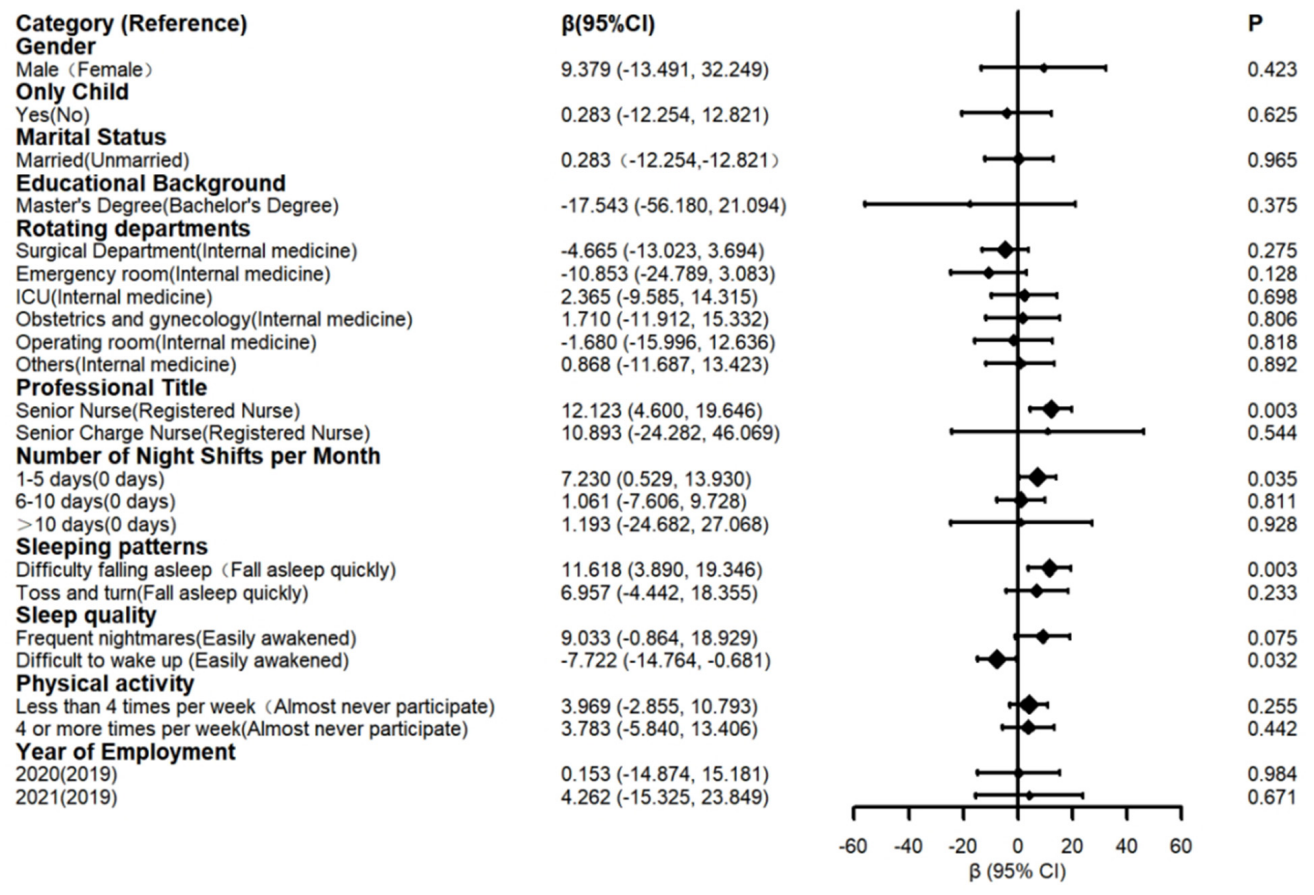
Linear mixed-effects model of factors affecting the mental health of new graduate nurses.

Figure 5 indicated that new graduate nurses with better sleep quality (less easily awakened, β = −7.72, *P* < 0.05) exhibited better mental health compared to those who were easily awakened. Additionally, new graduate nurses who had greater difficulty falling asleep showed poorer mental health than those who fell asleep quickly (β = 11.62, *P* < 0.05). The number of night shifts per month was also a significant factor affecting the mental health of new graduate nurses. Those who worked 1–5 night shifts per month had poorer mental health than those with no night shifts (β = 7.23, *P* < 0.05). Furthermore, new graduate nurses with the professional title of “senior nurse” reported worse mental health than those with the title of “nurse” (β = 12.65, *P* < 0.05).

## 4 Discussion

### 4.1 Overall and dimensional mental health status of new graduate nurses

The findings of this study indicate that the mental health status of new graduate nurses within their first 3 years of employment is relatively better compared to results reported in previous studies (16, 17). This may be attributed to the fact that Gansu Province is located in the inland northwestern region of China, where the economic development level lags behind that of coastal cities, and competitive pressure is relatively lower. New graduate nurses in this context may thus have access to certain career development opportunities, contributing to their relatively favorable mental health status.

However, when examined across different dimensions of mental health, new graduate nurses in this study showed higher scores on multiple SCL-90 subscales compared to other studies (18, 19). From T1 to T3, elevated scores were observed in dimensions such as obsessive-compulsive symptoms, depression, anxiety, and paranoid ideation. By the T3 time point, mean scores were high across all dimensions except for sleep and eating disorders, further suggesting that new graduate nurses have begun to exhibit a range of mental health issues.

### 4.2 Mental health trajectories and key time windows in new graduate nurses

It was observed that the mental health status of new graduate nurses tends to deteriorate progressively with increased work experience. Based on the observed trends in our data, we propose a tentative three-phase framework: a “high-pressure role adaptation period” during the first year, an “adaptation plateau period” from the first to second year, and a “career development confusion period” from the second to third year. This pattern may be partly attributed to the deepening transition from basic tasks to complex care, coupled with limited clinical experience (20), consistent with previous studies on new graduate nurses' transition challenges (10, 13). These dynamics may also intersect with broader workplace factors—such as hospital scheduling policies, unit culture, or staffing levels—that could shape the timing and intensity of each phase. In addition, selective attrition (e.g., nurses with greater distress leaving the workforce) may influence the observed trajectories and is also worth considering.

During the first year, new graduate nurses experience a significant decline in mental health—a phase we tentatively term the “high-pressure role adaptation period.” Faced with new environments, frequent rotations, and training demands, they endure substantial psychological pressure (21, 22). Similar to findings reported in prior research (10, 23), these challenges may be further shaped by organizational context, including orientation support and patient-to-nurse ratios, which could influence the adaptation process.

Entering the first to second year, mental health tends to stabilize temporarily, forming what we refer to as an “adaptation plateau period.” As nurses become familiar with workflows, stress may alleviate (24). Whether this stabilization reflects genuine psychological adjustment or is influenced by concurrent workplace changes (such as reduced rotation frequency or increased collegial support) remains to be explored in future studies.

Between the second and third years, mental health deteriorates again, entering the “career development confusion period.” Following rotation completion, nurses face independent responsibilities, career decisions, and family pressures (25). This decline may also relate to cumulative workplace stressors such as sustained understaffing or limited mentorship, rather than reflecting individual factors alone. It is important to emphasize that this three-phase framework is an exploratory interpretation based on our data, not a definitive developmental pattern. In line with recommendations from prior research, future studies incorporating organizational and contextual variables could help validate and refine this framework across diverse healthcare settings.

### 4.3 Obsessive-compulsive symptoms

Obsessive-compulsive symptoms (OCS), characterized by repetitive thoughts or behaviors performed to mitigate perceived risks, were the most elevated psychological dimension among new graduate nurses at all four time points (T0–T3), exceeding rates reported in similar urban studies in China (18, 19, 26). The healthcare setting necessitates strict adherence to protocols and aseptic techniques, which may reinforce checking and handwashing habits. Inexperience and fear of error likely further drive repetitive verification behaviors. For participants who underwent training during the COVID-19 pandemic (2020–2022), heightened hygiene protocols and infection control measures may have reinforced checking behaviors (27). However, despite consistently being the highest-scoring dimension at all time points (Table 2), OCS did not increase significantly over time (Figure 4). This pattern suggests that obsessive-compulsive tendencies may reflect relatively stable characteristics associated with the meticulous and rule-driven nature of nursing work, rather than progressive stress-induced symptoms. By contrast, somatization, anxiety, and depression showed clear worsening trends, indicating that these dimensions may be more responsive to accumulated work stress and therefore may represent key targets for intervention. The lack of significant change in OCS, despite high baseline levels, highlights the importance of distinguishing between stable occupational traits and stress-sensitive symptoms in mental health research.

### 4.4 Analysis of somatic symptoms and negative emotional states

During the 3-year follow-up period for new graduate nurses, somatic symptoms were particularly prominent. Their severity ranking rose significantly from the 7th position at the initial time point to the 4th, suggesting a pronounced somatization of psychological stress, commonly manifesting as headaches, sleep disturbances, and irregular eating patterns (28). Concurrently, negative emotions such as anxiety, depression, and psychotic symptoms also showed a clear upward trend (29, 30). As working hours increase and workload intensifies, new graduate nurses are more susceptible to decreased appetite, sleep problems, and emotional distress during their role adaptation process (10). Long-term exposure to high workload pressure has been associated with an increased risk of mental health disorders (23, 30). These associations, however, are derived from observational data and do not imply causality. Organizational factors—such as staffing levels, shift scheduling policies, or unit culture—may also contribute to these patterns and warrant further investigation. Nevertheless, the observed trends highlight the potential value of early attention to somatic and emotional symptoms in this population.

### 4.5 Factors associated with the mental health of new graduate nurses

The findings of this study reveal that sleep status and professional title are two significant factors associated with the mental health of new graduate nurses. Nurses with higher sleep quality and easier sleep initiation demonstrated significantly better mental health, suggesting that healthy sleep patterns have a protective effect on psychological wellbeing. However, given the observational nature of this study, we cannot rule out bidirectional effects, as psychological distress may also impair sleep. Extensive epidemiological studies have suggested a bidirectional interaction between sleep and mental health (31–33): insomnia and poor sleep quality are risk factors for psychological issues, while psychological stress can further impair sleep by disrupting HPA axis function and circadian rhythms (34). These dynamics may also be influenced by organizational factors such as shift scheduling policies, workload distribution, or workplace support systems, which were not directly examined in this study.

Additionally, new graduate nurses working 1–5 night shifts per month showed significantly worse mental health than those without night shifts, which may be associated with the disruption of biological rhythms and sleep deprivation caused by shift work (35, 36). Interestingly, this association was only significant for nurses working 1–5 night shifts per month, but not for higher frequencies. This non-linear pattern may reflect a threshold effect, where any night shift exposure increases risk, but the effect does not linearly increase with frequency; alternatively, nurses working more frequent night shifts may develop adaptive coping strategies over time, attenuating the psychological impact. Multiple studies have suggested that night shift work may be a key risk factor associated with significantly reduced sleep quality, and the deterioration of sleep quality may further contribute to mental health problems (37, 38). Therefore, it may be beneficial for nursing managers to consider prioritizing sleep issues and exploring interventions—such as optimized scheduling and early support—to address the potential cycle between sleep and mental health. However, interventional studies are needed to confirm the effectiveness of such strategies.

On the other hand, new graduate nurses with the professional title of “Senior Nurse” showed poorer mental health, which may stem from the higher expectations and pressures associated with this title (39). Nurse Practitioners are often assigned more complex clinical tasks, yet their actual experience and competence are still in the developmental stage. This mismatch between ability and expectations may contribute to self-doubt and anxiety (40, 41). From a practice perspective, this finding suggests that providing additional support (e.g., reasonable task allocation, mentorship) for this group during role transition may be valuable. Nevertheless, given the observational design, reverse causation cannot be ruled out—worsening mental health could theoretically affect career progression. Future research using cross-lagged panel designs may help clarify directionality.

## 5 Limitations

This study has the following limitations: First, the survey data was collected from a single city in northwest China. Given the differences in socio-cultural contexts and healthcare systems across regions, the mental health development trajectories of new graduate nurses during their role transition period may exhibit regional specificity. Therefore, the generalizability of the findings requires validation across broader geographical areas. Future research could further conduct cross-regional comparative analysis. Second, constrained by the study timeline, objective conditions, and the impact of the COVID-19 pandemic, the longitudinal sample size in this phase is limited. Currently, this dynamic cohort study is still ongoing; subsequent phases will expand the sample size and incorporate more relevant variables to enhance the study's explanatory power and robustness. Third, the SCL-90 lacks established thresholds for clinically meaningful change in nursing populations, limiting interpretation of the observed score differences. Fourth, our analysis focused on mean trajectories and did not explore inter-individual heterogeneity; future studies using latent class growth analysis could identify distinct trajectory subgroups and their predictors. Fifth, as with all observational research, causal inferences cannot be drawn from our findings; the associations reported should be interpreted with caution, and reverse causality or unmeasured confounding may exist.

## 6 Conclusion

This study tracked and explored the developmental trajectory of mental health status among new graduate nurses within their first 3 years of employment, as well as factors associated with these trajectories. The results indicate that the overall mental health level of new graduate nurses is acceptable at the initial stage of their careers, but shows a significant declining trend as their working years increase. Based on the observed patterns, we propose a tentative three-phase framework for understanding this trajectory: a “high-pressure role adaptation phase,” a “plateau phase,” and a “career development confusion phase,” with issues especially in somatization, anxiety, and depression. The study found that sleep quality, ease of falling asleep, frequency of night shifts, and professional title are key associated factors. These findings point to the potential value of phase-specific support for new nurses during their transition. From a practical perspective, optimizing shift schedules, promoting sleep health, and providing mentorship aligned with professional development stages may help alleviate psychological distress. Future research with larger and more diverse samples could further validate the proposed three-phase framework and explore the role of organizational factors in shaping mental health trajectories, offering additional insights for intervention development.

## Data Availability

The raw data supporting the conclusions of this article will be made available by the authors, without undue reservation.
