# Supplementary material for: Longitudinal study on the mental health trajectory of new graduate nurses during role transition: identifying critical windows and core influencing factors
Source: Front Public Health. 2026 Apr 9;14:1795005. doi: 10.3389/fpubh.2026.1795005 (PMC13104344; doi:10.3389/fpubh.2026.1795005)
Supplement: Supplementary file 1 [file Supplementary_file_1.docx]

Supplementary Table 4. Coefficients and Variance Inflation Factors (VIF) for All Predictors in the Linear Mixed-Effects Model

| Coefficients^a^ | | | | | | | |
| --- | --- | --- | --- | --- | --- | --- | --- |
| Variable | Unstandardized Coefficients | | Standardized Coefficients | *t* | *p* | Collinearity Statistics | |
|  | B | Std. Error | Beta |  |  | Tolerance | VIF |
| Constant | 115.589 | 8.622 |  | 13.407 | .000 |  |  |
| Time | 2.356 | 2.407 | .068 | .979 | .328 | .534 | 1.874 |
| **Sleep quality** |  |  |  |  |  |  |  |
| Frequent nightmares | 4.604 | 6.912 | .038 | .666 | .506 | .786 | 1.272 |
| Difficult to wake up | -15.481 | 4.673 | -.199 | -3.313 | .001 | .721 | 1.388 |
| **Sex**=Male | 7.901 | 7.572 | .062 | 1.043 | .298 | .734 | 1.362 |
| **Only Child**=Yes | -2.514 | 6.240 | -.021 | -.403 | .687 | .945 | 1.058 |
| **Educational Background**=Master's Degree | -22.791 | 14.784 | -.107 | -1.542 | .124 | .537 | 1.862 |
| **Marital Status**=Married | -12.818 | 9.171 | -.080 | -1.398 | .163 | .784 | 1.275 |
| **Rotating departments** |  |  |  |  |  |  |  |
| Surgical Department | 3.121 | 5.518 | .036 | .566 | .572 | .628 | 1.592 |
| Rotating Emergency room | 9.391 | 9.288 | .056 | 1.011 | .313 | .836 | 1.196 |
| ICU | 9.222 | 8.564 | .066 | 1.077 | .282 | .697 | 1.434 |
| Obstetrics and gynecology | 13.737 | 9.063 | .084 | 1.516 | .131 | .839 | 1.192 |
| Operating room | 2.146 | 7.000 | .020 | .307 | .759 | .590 | 1.695 |
| Others | 15.180 | 8.064 | .122 | 1.882 | .061 | .617 | 1.620 |
| **Physical activity** |  |  |  |  |  |  |  |
| Less than 4 times per week | 6.639 | 4.770 | .085 | 1.392 | .165 | .697 | 1.434 |
| 4 or more times per week | 7.254 | 6.860 | .065 | 1.057 | .291 | .686 | 1.459 |
| **Number of Night Shifts per month** |  |  |  |  |  |  |  |
| 1-5 days | 2.084 | 5.052 | .027 | .412 | .680 | .612 | 1.635 |
| 6-10 days | .320 | 6.110 | .004 | .052 | .958 | .557 | 1.797 |
| 10 days | -17.317 | 19.191 | -.048 | -.902 | .368 | .934 | 1.070 |
| **Sleeping patterns** |  |  |  |  |  |  |  |
| Difficulty falling asleep | 5.670 | 5.308 | .060 | 1.068 | .286 | .811 | 1.234 |
| Toss and turn | 9.804 | 7.718 | .069 | 1.270 | .205 | .886 | 1.128 |
| **Professional Title** |  |  |  |  |  |  |  |
| Senior Nurse | 14.278 | 5.752 | .170 | 2.482 | .014 | .555 | 1.801 |
| Senior Charge Nurse | 27.088 | 26.412 | .064 | 1.026 | .306 | .656 | 1.525 |

Note:**a,**Dependent variable is total SCL-90 score. All variance inflation factors (VIF) < 2, indicating no serious multicollinearity.

Supplementary Table 5. Baseline Characteristics by Completion Status (Among Participants with T0 Data, n=118)

| Variable | Complete Responders (n=88) | Incomplete Responders （n=30） | *p* |
| --- | --- | --- | --- |
| **SCL-90 total score, mean ± SD** | 120.10 ± 29.53 | 127.67 ± 49.67 | 0.436^a^ |
| **Sex,n (%)** |  |  | 0.562^b^ |
| Female | 79 (89.8) | 28 (93.3) |  |
| Male | 9 (10.2) | 79 (6.7) |  |
| **Educational Background,n (%)** |  |  | 0.306^b^ |
| Bachelor's Degree | 85 (96.6) | 30 (100) |  |
| Master's Degree | 3 (3.4) | 0 (0.0) |  |
| **Only Child,n (%)** |  |  | 0.773^b^ |
| No | 78 (88.6) | 26 (86.7) |  |
| Yes | 10 (11.4) | 4 (13.3) |  |
| **Marital Status,n (%)** |  |  | -^c^ |
| Unmarried | 88 (100.0) | 30 (100.0) |  |
| Married | 0 (0.0) | 0 (0.0) |  |
| **Professional Title,n (%)** |  |  | 0.613^b^ |
| Registered Nurse | 83 (94.3) | 29 (96.7) |  |
| Senior Nurse | 5 (5.7) | 1 (3.3) |  |
| Senior Charge Nurse | 0 (0.0) | 0 (0.0) |  |
| **Number of Night Shifts per Month,n (%)** |  |  | 0.456^b^ |
| 0 days | 36 (40.9) | 16 (53.3) |  |
| 1-5 days | 35 (39.8) | 11 (36.7) |  |
| 6-10 days | 15 (17.0) | 2 (6.7) |  |
| ＞10 days | 2 (2.3) | 1 (3.3) |  |
| **Sleeping patterns,n (%)** |  |  | 0.862^b^ |
| Fall asleep quickly | 70 (79.5) | 24 (80.0) |  |
| Difficulty falling asleep | 13 (14.8) | 5 (16.7) |  |
| Toss and turn | 5 (5.7) | 1 (3.3) |  |
| **Sleep quality,n (%)** |  |  | 0.677^b^ |
| Difficult to wake up | 55 (62.5) | 18 (60.0) |  |
| Frequent nightmares | 7 (8.0) | 4 (13.3) |  |
| Easily awakened | 26 (29.5) | 8 (26.7) |  |
| **Physical activity,n (%)** |  |  | 0.169^b^ |
| Almost never participate | 18 (20.5) | 7 (23.3) |  |
| Less than 4 times per week | 50 (56.8) | 19 (63.3) |  |
| 4 or more times per week | 20 (22.7) | 4 (13.3) |  |
| **Rotating departments,n (%)** |  |  | 0.634^b^ |
| Internal medicine | 15 (17.0) | 6 (20.0) |  |
| Surgical Department | 22 (25.0) | 10 (33.3) |  |
| Emergency room | 8 (9.1) | 1 (3.3) |  |
| ICU | 10 (11.4) | 1 (3.3) |  |
| Obstetrics and gynecology | 7 (8.0) | 2 (6.7) |  |
| Operating room | 15 (17.0) | 4 (13.3) |  |
| Others | 11 (12.5) | 6 (20.0) |  |

Note:All *p*> 0.05, indicating no significant differences between completers and non-completers at baseline.

a, Independent t-test (unequal variances assumed, corrected result). b, Pearson's chi-square test. c, Not computed because marital status was constant (all unmarried).

Supplementary Table 6. Comparison of First Available Measures Between Participants With and Without T0 Data

| Variable | Complete Responders (n=88) | Incomplete Responders （n=30） | *p* |
| --- | --- | --- | --- |
| **SCL-90 total score, mean ± SD** | 122.03 ± 35.65 | 135.42 ± 40.49 | 0.223^a^ |
| **Sex,n (%)** |  |  | 0.419^b^ |
| Female | 107 (90.7)) | 10 (83.3) |  |
| Male | 11 (9.3) | 2 (16.7) |  |
| **Educational Background,n (%)** |  |  | 0.067^c^ |
| Bachelor's Degree | 115 (97.5) | 10 (83.3) |  |
| Master's Degree | 3 (2.5) | 2 (16.7) |  |
| **Only Child,n (%)** |  |  | 0.629^b^ |
| No | 104 (88.1) | 10 (83.3) |  |
| Yes | 14 (11.9) | 2 (16.7) |  |
| **Marital Status,n (%)** |  |  | -^c^ |
| Unmarried | 118 (100.0) | 12 (100.0) |  |
| Married | 0 (0.0) | 0 (0.0) |  |
| **Professional Title,n (%)** |  |  | **<0.001^b^** |
| Registered Nurse | 112 (94.9) | 6 (50.0) |  |
| Senior Nurse | 6 (5.1) | 6 (50.0) |  |
| Senior Charge Nurse | 0 (0.0) | 0 (0.0) |  |
| **Number of Night Shifts per Month,n (%)** |  |  | 0.241^b^ |
| 0 days | 52 (44.1) | 2 (16.7) |  |
| 1-5 days | 46 (39.0) | 7 (58.3) |  |
| 6-10 days | 17 (14.4) | 2 (16.7) |  |
| ＞10 days | 3 (2.5) | 1 (8.3) |  |
| **Sleeping patterns,n (%)** |  |  | 0.228^b^ |
| Fall asleep quickly | 94 (79.7) | 7 (58.3) |  |
| Difficulty falling asleep | 18 (15.3) | 4 (33.3) |  |
| Toss and turn | 6 (5.1) | 1 (8.3) |  |
| **Sleep quality,n (%)** |  |  | 0.641^b^ |
| Difficult to wake up | 34 (28.8) | 2 (16.7) |  |
| Frequent nightmares | 11 (9.3) | 1 (8.3) |  |
| Easily awakened | 73 (61.9) | 9 (75.0) |  |
| **Physical activity,n (%)** |  |  | 0.974^b^ |
| Almost never participate | 25 (21.2) | 3 (25.0) |  |
| Less than 4 times per week | 69 (58.5) | 7 (58.3) |  |
| 4 or more times per week | 24 (20.3) | 2 (16.7) |  |
| **Rotating departments,n (%)** |  |  | 0.794^b^ |
| Internal medicine | 21 (17.8) | 1 (8.3) |  |
| Surgical Department | 32 (27.1) | 5 (41.7) |  |
| Emergency room | 9 (7.6) | 1 (8.3) |  |
| ICU | 11 (9.3) | 1 (8.3) |  |
| Obstetrics and gynecology | 9 (7.6) | 0 (0.0) |  |
| Operating room | 19 (16.1) | 3 (25.0) |  |
| Others | 17 (14.4) | 1 (8.3) |  |

Note:No significant difference in the first measured SCL-90 scores was found between the two groups, supporting the comparability of participants with and without T0 data.

a,Independent t-test. b,Pearson's chi-square test. c,Fisher's exact test. d,Not computed because marital status was constant (all unmarried).

Supplementary Table 7. Paired t-Test Comparing SCL-90 Total Scores Between T1 and T2 Among Complete Responders (n=88)

| Time | Mean | SD | Mean Difference | 95% CI | *t* | df | *p* |
| --- | --- | --- | --- | --- | --- | --- | --- |
| T1 | 129.74 | 33.79 | 0.01 | [-6.19, 6.22] | 0.004 | 87 | 0.997 |
| T2 | 129.73 | 40.43 | 129.73 |  |  |  |  |

Note: Paired t-test results show no significant difference between T1 and T2 scores, indicating that the observed decrease in Figure 1 is not statistically meaningful.


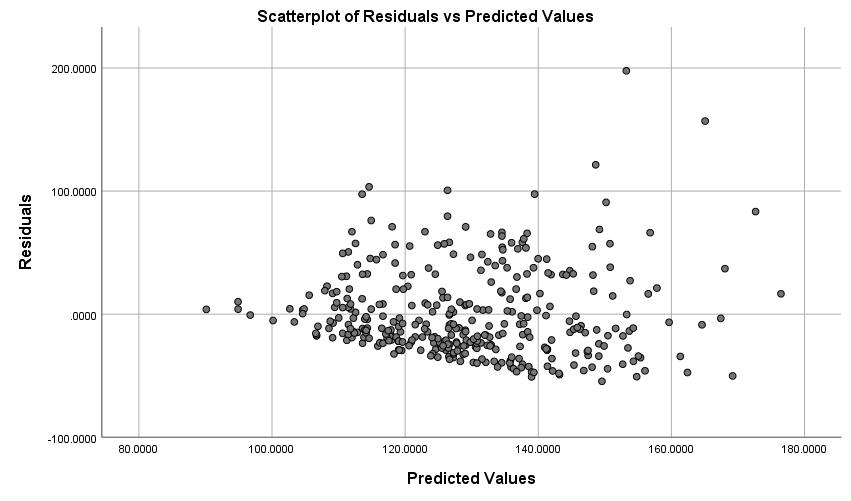


Supplementary Figure 5. Scatterplot of residuals versus predicted values.

The random distribution of points around zero suggests no violation of linearity or homoscedasticity.


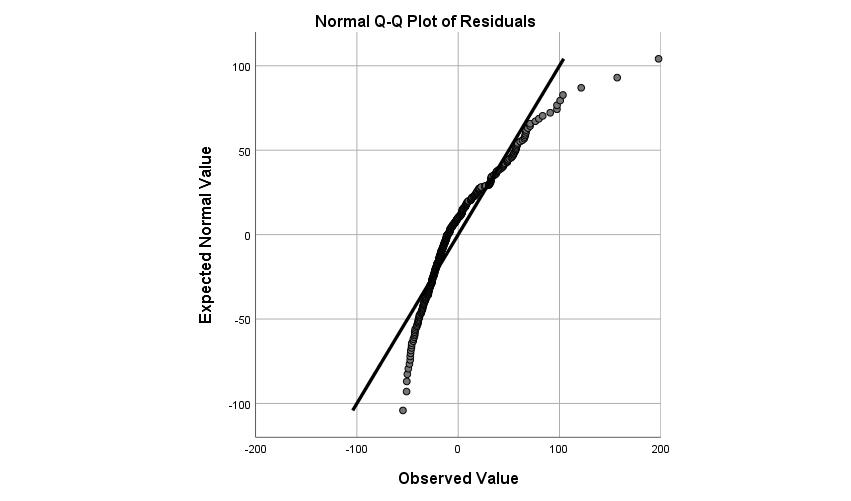


Supplementary Figure 6. Normal Q-Q Plot of Residuals

Most points fall close to the diagonal line, indicating approximate normality.


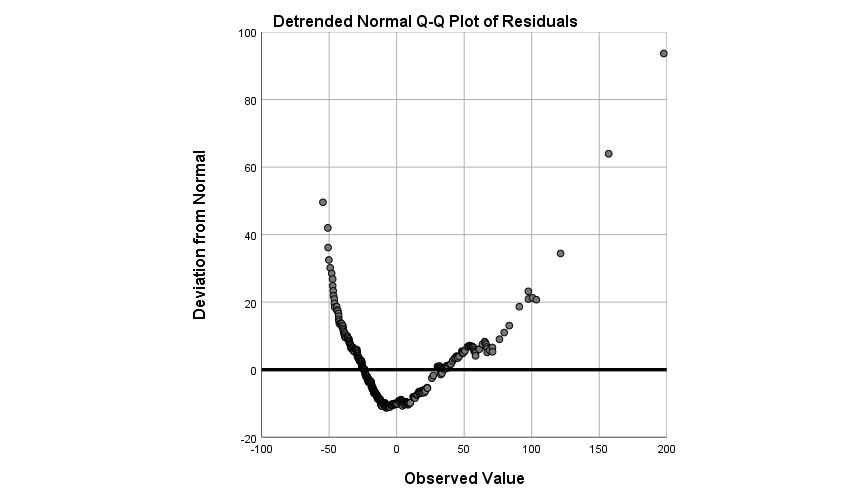


Supplementary Figure 7. Detrended normal Q-Q plot of residuals.

Points are randomly scattered around zero, further supporting the normality assumption.
